# Mental Well-Being and Job Satisfaction of Hospital Physicians during COVID-19: Relationships with Efficacy Beliefs, Organizational Support, and Organizational Non-Technical Skills

**DOI:** 10.3390/ijerph19063734

**Published:** 2022-03-21

**Authors:** Vincenza Capone, Roberta Borrelli, Leda Marino, Giovanni Schettino

**Affiliations:** Department of Humanities, University of Naples Federico II, 83100 Naples, Italy; roberta.borrelli993@gmail.com (R.B.); leda.marino@unina.it (L.M.); giovanni.schettino@unina.it (G.S.)

**Keywords:** physicians’ well-being, efficacy beliefs, organizational support, non-technical skills, job satisfaction

## Abstract

The COVID-19 outbreak has led worldwide governments to take preventive measures to contain the spread of the virus and its extraordinary demands upon healthcare workers. Consequently, healthcare workers have been under high pressures, putting them at risk of developing adverse outcomes. The present study aims to investigate the psychological and organizational factors that contributed to physicians’ well-being during the pandemic. A total of 78 Italian physicians participated in the study. They completed a self-report questionnaire measuring efficacy beliefs, orientation towards patient engagement, job satisfaction, non-technical skills, organizational support, sense of belonging to the hospital, job satisfaction, and mental well-being. Physicians’ sense of belonging to their hospital, efficacy beliefs about their organizations and communication with patients, as well as non-technical skills related to communication and risk awareness were positively associated with job satisfaction. In addition, the latter and sense of belonging to own hospital were positively associated with mental well-being. These findings may guide policymakers and healthcare organizations managers to consider the potential psychosocial factors related to physicians’ well-being and the required preventive measures that can help in enhancing their human and organizational resources to cope with stressful situations such as the COVID-19 pandemic.

## 1. Introduction

Medical practice is defined as a profession with high demands, suggesting that these are likely to have a long-term negative impact on worker lifestyle and result in psychological problems [1]. During the SARS-CoV-2 pandemic, medical healthcare workers were under an enormous amount of workload pressure along with increased total health expenditures [2]). As highlighted in a recent review [3], with the onset of COVID-19, increasing occupational hazards and personal stressors caused further disruption. In support, recent studies demonstrated that the COVID-19 pandemic can be regarded as a collective traumatic event [4]. During the first phases of the pandemic, physicians worked long hours and were exposed to fatigue and other stressors such as fear of infection, putting their well-being at risk [5]. In addition, experiencing repeated loss of life, threat to one’s own life, fatigue, and isolation, and the loss of daily routine and grounding rituals, resulted in sleep disturbances, anxiety, and depression [3].

Despite the increased attention and resources that have been directed toward healthcare workers’ burnout, stress, anxiety symptoms, and depression [6,7]; physician well-being has remained under guarded conditions [8].

The impact of the pandemic on the National Health System has made it necessary to analyze the psychosocial and organizational fallout that the emergency has had for healthcare organizations exposed on the front lines, and the potential consequences on practitioners’ well-being. COVID-19 is likely to continue to result in negative effects in the future; thus, it is important to keep doctors physically, mentally, and emotionally supported throughout the pandemic. In this regard, recent calls to action mandated the need to provide high-quality data on the psychological impacts of the COVID-19 pandemic [8,9]. To date, little research has analyzed the levels of hospital physicians’ mental well-being at work in a positive key [10] during the pandemic, and in terms of the psychological and organizational resources related to it. From our perspective [8], analyzing malaise is a necessary but not sufficient condition to consider the potential resources of the individual at work and their consequences. Furthermore, as it has been pointed out by Rudolph and colleagues [11], focusing on well-being issues should be differentiated according to occupational group. Thus, it is a priority to understand the psychological needs of health workers in order to provide them with the appropriate tools to mitigate the negative effects of dealing with the pandemic [12].

On these grounds, the present work aimed to investigate the well-being, operationalized as mental well-being and job satisfaction, of hospital physicians during the acute phase of the pandemic, and the relationships with some promoting factors, such as personal and organizational beliefs, orientation towards patient engagement, and organizational support.

Compared to other jobs, which during the pandemic emergency were able to activate remote work protocols, the hospitalists have always worked in hospital, risking their physical and psychological safety. From an organizational perspective, this also required hospital organizations to allocate resources to ensure the safety of people. In addition to the application of “technical” guidelines [13], it is fundamental considering psychosocial safety and “non-technical skills” have been outlined in other settings [14], upon which both the effective application of technical measures and their perceived psychological safety and well-being depend [15]. Therefore, a further objective of this work was to investigate the relationship between perceptions of non-technical skills in the organization and the well-being of hospital physicians.

### 1.1. Mental Well-Being and Job Satisfaction at Work

The World Health Organization (WHO) defines an individual’s mental health as a “state of well-being in which the individual realizes his or her abilities, can cope with the normal stresses of life, can work productively and fruitfully, and is able to make a contribution to his or her community” [9] (p. 60). This is in line with a Positive Psychology perspective: well-being and malaise are not two opposite poles.

Consequently, the conceptualizations of well-being at individual levels can be categorized on two dimensions [10,16,17]: well-being as a context-free (e.g., general mental health) and as a domain-specific concept (e.g., job satisfaction, work engagement). Following this, literature suggested that considering the two conceptualizations of well-being was preferable [18].

When well-being was examined as a domain-specific concept, the associations with its antecedents were stronger [17]. Thus, we intend to consider, on the one side the mental well-being as a positive individual outcome for the life of workers and, on the other side, job satisfaction as a crucial organizational outcome.

Regarding mental well-being, Keyes [10,18] takes up the concept of WHO mental health and argues that mental health is a state of complete emotional, psychological, and social well-being which does not match, therefore, the simple absence of psychopathology [19]. In the Mental Health Continuum Model [10,19,20], mental health is regarded as a syndrome of symptoms of positive feelings and positive functioning in life.

It includes three domains: emotional, psychological, and social well-being. A comprehensive assessment of an individual’s positive mental health and well-being, therefore, requires considering all these dimensions: subjective well-being (feeling good and satisfied), psychological well-being (interpersonal and intrapersonal functioning), and social well-being (sense of belonging to a community and making a contribution to society) [10,21]. As a relevant part of an individual’s life, work experience can also affect people’s perceived well-being [22]. Thus, the happy-productive worker’s hypothesis has often been examined in organizational research by correlating job satisfaction and psychological well-being [23].

Specifically, recent studies [24] consider that workers’ well-being plays an important role in job satisfaction and their subsequent retention, especially when employees perceive positive emotions in the workplace.

In this regard, job satisfaction represents one of the most immediate effects of the organization on individuals at an emotional and cognitive level [25], and it is recognized as a good predictor of absenteeism [26] and turnover intention [27]. Job satisfaction can be regarded as a psychological construct that refers to a person’s responses to their job. Locke’s [28] definition, which is most widely accepted in the literature [29,30], states that job satisfaction is a “pleasurable or positive emotional state resulting from the appraisal of one’s job” [28] (p. 1304). Concerning the healthcare sector, the literature has widely highlighted that high physician job satisfaction benefits physicians’ physical and mental health [31].

Indeed, it can be a protective factor against burnout, intention to leave, absenteeism [26], and turnover intention [32,33].

Healthcare professionals’ satisfaction is an important service quality marker as well as a work resources issue that affects their psychological well-being [34]. Some Italian studies [31,32,33,34,35] documented a relationship between health professionals’ well-being and variables such as job satisfaction, personal and collective efficacy, and sense of belonging to the organization.

In line with the literature [36] job satisfaction and well-being are two related constructs influenced by organizational efficacy, a subcategory of collective efficacy [35].

### 1.2. Physicians’ Efficacy Beliefs and Orientation towards Patient Engagement

The COVID-19 pandemic and the recommendations of social distancing and home isolation to limit the spread of the virus have significantly transformed relationships among doctors and patients. It is highly likely that changes in how doctors delivered their services would have impacted the effectiveness of the doctor-patient interaction [37].

Previous studies have demonstrated that physicians’ communication skills improved patients’ quality of life [38] with a positive impact both on patients’ and doctors’ satisfaction [39]. Indeed, physicians with better communication skills were more likely to inquire about patients’ concerns, set goals for successful treatment [40], and offer better emotional support [41]. Moreover, recent studies [42] affirm that promoting a patient-centered communication, through the improvement of physicians’ communication skills can also reduce healthcare costs. On the subject and from a social cognitive perspective [43], perceptions of communication efficacy resulted as predictors of well-being and job satisfaction in health professionals [18,19,20,21,22,23,24,25,26,27,28,29,30,31,32,33,34,35,36,37,38,39,40,41,42,43,44], also promoting professional self-realization [45].

In support, during the pandemic, Messerotti and colleagues [46] documented a negative association between physician communication skills and burnout, which, in turn, was negatively related to physicians addressing patients’ emotions with empathy and fostering shared decision making.

Due to the centrality of the patient in the process of care and in the evaluation of the organization’s quality standards [47], taking into account communication with patients means rethinking their centrality in the care pathway in line with what the patient engagement advocates [48]. Healthcare professionals’ orientation towards psychosocial needs and the engagement of patients is a protective factor against emotional exhaustion [49].

From this perspective, however, the Health Engagement framework sustains that the low levels of communication skills of doctors and patients, also considered as “citizens”, can inhibit the health engagement process [48] (p. 36). Even more, in the pandemic scenario, the literature [50] has shown the crucial role of collaboration between citizens and healthcare professionals supporting the argument that citizens’ engagement in health management and their attitudes toward preventive measures are functions of the individual’s efficacy beliefs.

Therefore, it is plausible to suppose a positive association between physicians’ communication self-efficacy, their orientation toward patient engagement, and well-being. Examining these relationships is of greater importance in a health emergency, such as that related to COVID-19, where healthcare professionals play a key role in communicating the advance of the crisis and the behaviors to adopt.

Hospitals involve a complex socio-technical health system where working groups influence the quality of the organization and contribute to adverse clinical events and outcomes [22]. In health organizations, being part of a successful team, in which members support each other, promotes health professionals’ well-being, organization commitment, and willingness to provide services that improve patients’ satisfaction [51].

While self-efficacy refers to an individual’s confidence in their abilities, collective efficacy consists of employees’ beliefs about how well their group can perform and how well they can coordinate efforts to achieve organizational goals [52].

Collective efficacy beliefs are a significant component in predicting psychological well-being for individuals [53]. As confirmed by Salanova et al. [54], working groups under time pressure with low collective efficacy report an increase in collective anxiety.

In contrast, collective efficacy beliefs are positively associated with self-efficacy [55] and represent an essential predictor of performance [56], and psychological well-being [53]. Furthermore, in such beliefs an antecedent of people’s sense of belonging to their organization [57] and organizational support [58] can be identified. In this respect, recent studies [59] highlight the positive role of organizational support on well-being and job satisfaction.

### 1.3. Perceived Organizational Support

Healthcare organizations have begun to reflect on how working conditions can contribute to well-being or malaise and directly affect the occupational health status of healthcare workers [60,61,62].

In the organizational setting, positive relationships with others would tend to reinforce conditions of well-being [63], whereas the absence of support would fuel conditions of distress [64,65,66,67]. In the pandemic era, organizational support emerged as fundamental for healthcare workers. It is a job resource that can improve employees’ resources such as self-efficacy, which, in turn, may lead to positive psychological and organizational outcomes [68].

When people receive organizational support, they show more positive behaviors towards their organization and become more engaged to offer their ideas to their organization [69].

A large body of research [70,71,72,73] documented that perceived organizational support—i.e., employees’ “global beliefs concerning the extent to which the organization values their contributions and cares about their well-being” [74] (p. 501)—is positively associated with various facets of well-being.

In view of that, exploring the relationship between these variables and the well-being of healthcare workers during the pandemic could provide important insights into their mental state [75]. In addition, literature [68,69] also shows strong relationships between organizational support and job satisfaction and their “buffering” role in malaise outcomes among frontline workers during COVID-19 [8].

### 1.4. Perceived Safety Related to COVID-19 Contagion and Specific Organizational Skills

Workers’ perceptions of safety in their organizations concerning COVID-19 risks are also related to Non-Technical Skills (NTS) [15].

The latter may be conceived as “the cognitive, social and personal resources skills that complement technical skills, and contribute to safe and efficient task performance” [76] (p. 1). “The perception of safety of workers can be expressed in four dimensions that qualify the organization capacity to manage the return to work during the COVID-19 pandemic, namely situational awareness, capacity to communicate and make decisions effectively and efficiently, and the capacity to recognize additional mental and physical fatigue, generated by the pressure of working in the presence of an “invisible enemy” and by the stress of having to use devices and measures necessary to contain the contagion risk.” [76] (p. 9). Consequently, NTS represents a determinant key of workplace safety [15].

In this regard, the literature has investigated the link between NTS and safety performances across different working sectors [15,77].

In the organizational sector, Converso et al. [15] highlight the crucial role of the organizational barriers and facilitators in promoting or inhibiting NTS required to ensure the safety of work practices. In the pandemic context, healthcare workers who have faced a biological risk and/or received specific training evaluate safety at work by assessing not only their performances but also the organizational heed to the use of NTS [15].

It is possible to consider the framework of NTS as a core of resources [78] that play a buffering role in contrast to job demand pressures [79].

NTS are specific systems to the workplace that refer to the perceived quality and effectiveness of procedures and interventions implemented by an organization to improve safety outcomes concerning the COVID-19 risk. From this perspective, NTS reflect the ability of the whole organization and its members to adopt and support specific behaviors concerning the risk of the COVID-19 disease:

- Communication is a crucial process for teamwork and workplace safety that refers to the exchange of information, feedback or possible reactions concerning the COVID-19 risk;
- Decision making allows one to choose the best possible option in a specific situation;
- Situational awareness enables constant monitoring in the workplace, identifying possible relevant changes in the workplace regarding the COVID-19 risk;
- Fatigue management enables one to detect antecedents and consequences of fatigue (mental and physical) related to protective behaviors at work as well as the implementation of coping strategies;
- Personal contribution encourages employees’ contribution to organizational decisions [78] (pp. 5–7).

From a theoretical point of view, we could conceive NTS as complementary to technical skills because both contribute to a safe and efficient performance [80].

As job resources are functional in achieving work goals, in terms of both productivity and safety outcomes, NTS may also reduce the perceived risk of being infected at work and the associated psychological/physiological costs. In line with this conceptualization [78] previous studies [81,82] demonstrated that psychological safety climate, job resources related to safety at work (e.g., knowledge, social support) could reduce malaise outcomes and could promote well-being and job satisfaction through positive job resources, which play a buffering role in well-being. In this scenario, a new perspective is coming: which is the role of the organization regarding the support of safety?

### 1.5. Aim of the Study

Well-being, health, and quality of life in work environments have become important issues in healthcare management [83,84].

Following this line of reasoning, we aimed to examine the relationships between physicians’ well-being, efficacy beliefs both related to their communication with patients and organization, non-technical skills, organizational support, engagement orientation, and sense of belonging to their organization. Specifically, the present study aimed to test the following hypotheses:

**Hypothesis** **1.** *Collective efficacy, communication self-efficacy, orientation toward patient engagement, sense of belonging and non-technical skills were positively associated with job satisfaction.*

**Hypothesis** **2.** *Collective efficacy, communication self-efficacy, orientation towards patient engagement, sense of belonging, organizational support, and job satisfaction were positively associated with mental well-being.*

## 2. Material and Methods

### 2.1. Participants

Participants consisted of 78 Italian physicians of different medical specializations (response rate 25% among them: 11.5% were anesthetists; 8.9% were cardiologists; 14% were surgeons, general or specific; 6.4% were hematologists; 5% were emergency room doctors; 8.9% were internal medicine doctors; 6.4% were transfusion medicine doctors; 5% were nephrologists; 8.9% were orthopedists; 2.5% were psychiatrists; 7.6% were radiologists).

Physicians ranged from 24 to 65 years of age (M = 46.5, SD = 12.15), and 66.7% of the sample was male. Of them, 67.9% were First Level Medical Managers, 19.2% were Service Managers, and 12.8% were Second Level Medical Managers. Overall, participants had 16 years of service (M = 16.4, SD = 12.13), with a mean of 8.89 years of work in the same hospital (SD = 9.53) and a mean of 8.88 years in the same hospital ward (SD = 9.19). Physicians had an average of 40 working hours per week (M = 40.5, SD = 9.18; Range 9–66).

### 2.2. Procedure

A cross-sectional study was performed during the third wave of the Coronavirus outbreaks (December 2020–May 2021). Using GPower 3.1, we estimated the required sample size for detecting a small-sized effect (d = 0.15) with an alpha = 0.05, power = 0.80, and six predictors. The estimated sample size was N = 55 for multiple regression. Hence, we invited 100 physicians, and 78 of them took part in the research. They voluntarily completed an online self-report questionnaire on the Google Forms platform, which took approximately 15 min to complete. The sample was recruited through a convenience sampling strategy. In particular, the first author contacted two head doctors of two big hospitals in Campania (a south Italy region) who were introduced to the research project and its aims, its methodology, the questionnaire that had been developed for testing the hypotheses, and its data collection procedure, and asked for the involvement of doctors through internal communication channels (i.e., e-mails, bulletin board messages).

Participation was anonymous, no incentive was given, and written informed consent was obtained from all participants through a specific section in the questionnaire. All procedures followed were in accordance with the ethical standards and the Helsinki Declaration of 1975, as revised in 2000. All participants were given the option to withdraw at any moment.

### 2.3. Measures

The survey instrument was a self-report questionnaire. The latest included a socio-demographic section that included questions about physicians’ careers (i.e., type of medical specializations or how many hours per week they spent in work activities).

Moreover, psychological variables in the study were measured using the following Likert scales.

#### 2.3.1. Collective Efficacy Scale for Producers’ Organizations

The workers’ beliefs about their ability to successfully cope with critical situations related to their role [85] were assessed by the *Collective Efficacy Scale for Producers’ Organizations* [86]. The tool consists of six evaluations with a five-point Likert scale ranging from one (very disagree) to seven (very agree). An example item is “I am convinced that I always live up to the responsibilities assigned to me”. The alpha internal reliability coefficient for this scale was 0.77.

#### 2.3.2. Physicians’ Communication Perceived Self-Efficacy

The physicians’ beliefs related to their ability to successfully manage problematic situations referring to patient communication [18] were assessed by the *Scale of physicians’ communication self-efficacy short form* [87].

The tool consists of eight items evaluated with a five-point Likert scale ranging from one (not at all capable) to five (fully capable). An example item is “How capable do you feel you are of asking the patient about his or her concerns concerning the condition from which he or she suffers?”. The alpha internal reliability coefficient for this scale was 0.86.

#### 2.3.3. Health Professionals’ Orientation towards Patient Engagement

In order to detect the participants’ orientation toward patient engagement during the pandemic, we used the *Scale of healthcare workers’ orientation towards patient engagement* [88]. The tool consists of 12 items evaluated with a six-point Likert scale ranging from one (totally disagree) to six (totally agree).

An example item is “The patient’s active role in preventing or mitigating COVID-19-related symptoms is critical”. The alpha internal reliability coefficient for this scale was 0.76.

#### 2.3.4. Sense of Belonging to Hospital

The physicians’ sense of belonging to their hospital and others (i.e., colleagues) was detected by the *Scale of Sense of Belonging* [31], consisting of three items evaluated with a four-point Likert scale ranging from one (strongly disagree) to four (strongly agree).

An example item is “I feel that I belong to this hospital”. The alpha internal reliability coefficient for this scale was 0.88.

#### 2.3.5. Organizational Support

The perceived support received from colleagues and hospital management during the COVID-19 pandemic was detected by two items ad hoc [22] rated on a ten-point Likert scale ranging from one (not at all) to ten (totally).

An example of an item is “How much do you feel you can count on the support of your colleagues in this COVID-19 emergency phase?”. The alpha internal reliability coefficient for this scale was 0.76.

#### 2.3.6. Non-Technical Skills

The physicians’ non-technical skills during the COVID-19 pandemic were assessed by the *SAPH@W Questionnaire* [15]. The tool consists of 20 items and contains five dimensions to explore the job safety perceived by physicians during the Coronavirus outbreak: situational awareness (e.g., “Identify specific contagion risk by COVID-19 in your job”; α = 0.91), decision-making (e.g., “Make quick decisions”; α = 0.94), communication (e.g., “Communicate effectively with the supervisor on risks related to COVID-19”; α = 0.86), fatigue management (e.g., “Adopt measures to reduce mental fatigue due to such behaviours”; α = 0.97) and personal contribution (e.g., “understand the situation regarding contagion risks α = 0.90).

The subscales are evaluated with a five-point Likert scale ranging from one (not at all) to five (completely).

#### 2.3.7. Job Satisfaction

The participants’ job satisfaction was detected by a *single item* (“What is your level of satisfaction with your job?”), according to Cortese and Quaglino [89]. The item is rated on a seven-point Likert scale ranging from one (I am extremely dissatisfied) to seven (I am extremely satisfied).

#### 2.3.8. Mental Well-Being

Physicians’ mental well-being was detected by the *Italian Mental Health Continuum-Short Form* [90]. The tool consists of 14 items evaluated with a six-point Likert scale ranging from one (never) to six (every day) and measures emotional well-being (EWB; α = 0.88), psychological well-being (PWB; α = 0.88) and social well-being (SWB; α = 0.80).

An example item is “During the past month, how often did you feel happy?”. The alpha internal reliability coefficient for the full scale was 0.91.

### 2.4. Statistical Analyses

Descriptive statistics were used to summarize variables collected in the questionnaire and the main characteristics of the study population. According to Vaske et al. [91], alpha values between 0.65 and 0.80 are considered “adequate” for scales adopted for research on human dimensions. Regarding the scores of the scales adopted in the questionnaire, the average of the item scores was considered. In addition, Pearson correlation was performed to analyze the association between variables. Data analysis was performed using IBM SPSS Statistics 27.0. Since the exiguous sample was used, different linear regressions were carried out to test the hypothesized relationships.

## 3. Results

### 3.1. Descriptive Analyses

Descriptive analysis in Table 1 showed that the sample reported above-average levels of mental well-being, particularly in the psychological dimension (M = 4.01; SD = 0.98).

Concerning organization variables, participants showed high levels of job satisfaction (M = 4.91; SD = 1.41), collective efficacy (M = 5.35; SD = 0.90), perceived support from their organization (M = 6.31; SD = 1.92), and sense of belonging to their hospital (M = 2.74; SD = 0.63). About the communication area, they reported the high efficacy and effectiveness of their organizations in managing the communication on COVID-19 (M = 3.53; SD = 0.91). Furthermore, the physicians feel very capable of communicating with patients (M = 3.39; SD = 0.61) and taking into account their active participation in care-related decision-making (M = 4.30; SD = 0.69). With regard to the pandemic situation, although respondents declared their organization was very aware of contagion risk, they believed their hospital employees were unable to endure the fatigue linked to adopting preventive measures of the contagion (M = 2.46; SD = 1.10).

### 3.2. Correlations

In Table 2, the correlations among the variables are displayed. Physicians’ well-being was positively correlated with job satisfaction, collective efficacy, sense of belonging to their organization, and non-technical skills about communication and decision-making, but not with other dimensions of non-technical skills and the orientation towards patient engagement. Communication self-efficacy was positively correlated with the psychological dimension of well-being only, collective efficacy, and orientation towards patient engagement, which in turn was positively associated with collective efficacy beliefs, non-technical skills related both to collective ability in making decisions to prevent contagion risk by COVID-19 and participants’ personal contribution in coping with the pandemic in their workplace. Moreover, job satisfaction was positively correlated with collective efficacy, non-technical skills related to communication and decision-making, and sense of belonging to their own organization. In addition, the latter was positively associated with organizational support and the different dimensions of non-technical skills. Finally, perceived support from own organization was positively correlated with the perceived collective efficacy and social dimension of well-being.

### 3.3. Multiple Regression Analyses

Multiple regression analyses were run to test the hypothesized relationships between the psychological variables (Table 3, Table 4, Table 5 and Table 6) included in the study. As displayed in Table 3, Hypothesis 1 was partially confirmed. Findings indicated that physicians’ efficacy beliefs and sense of belonging to their hospital were positively associated with job satisfaction (R^2^ = 0.374; *p* = < 0.05). In contrast, organizational support and participants’ orientation towards patient engagement were not significantly associated with the satisfaction for their job. Moreover, we added job satisfaction in Model 2 to test the association with mental well-being as hypothesized (Hypothesis 2). In this respect, results identified job satisfaction and sense of belonging as two important variables for physicians’ mental health (R^2^ = 0.172; *p* < 0.05). Regarding non-technical skills relationships with job satisfaction, only those related to risk awareness resulted associated with job satisfaction (R^2^ = 0.114; *p* < 0.05). Finally, we included job satisfaction in Model 4 to test Hypothesis 2. Findings revealed that job satisfaction was only significantly and strongly related to mental well-being (R^2^ = 0.285; *p* < 0.001).

## 4. Discussion

Our study aimed to examine the relationships between physicians’ well-being and job satisfaction and some individual and organizational variables, such as efficacy beliefs, non-technical skills, and organizational support during the pandemic.

The findings showed that, despite the COVID-19 emergency, the levels of psychosocial well-being experienced by hospital physicians were high, as are those of job satisfaction. This is perhaps an unexpected finding, as well-being was not as low as might be perceived by the general public, but is in line with studies conducted in the UK in the first wave of the pandemic [92]. It is likely due to the social recognition that physicians received during the pandemic and also the increased support from institutions due to coping with the health emergency. Further studies, possibly longitudinal, should be conducted in the future. It will be necessary to understand, in the long term, the impact of a prolonged pandemic. Our study is a useful starting point: it reflects a snapshot of the moment and brings attention to some correlates of physician well-being during the pandemic.

Physicians score quite highly on the other variables and, in particular, on organizational support and all dimensions of non-technical skills, defined as those skills that are not specific but can contribute to the activation of a more effective and safer performance.

Furthermore, our results, consistent with pre-pandemic works [31], emphasized the role of perceptions of efficacy in well-being at work.

Despite the emergency and the limited time available due to the overflow of requests for assistance, physicians still reported feeling quite capable of communicating effectively with patients and being helpful to them. Communication self-efficacy beliefs, as evidenced in the literature, played a key role in physicians’ perceptions of well-being. The relationship with the patient, considered the center of the care process and the ultimate judge of the quality of care provided, is reported by several studies as a source of stress and anxiety [35,93] due to a lack of training about communication skills that make hospital physicians feel unable to communicate effectively with users [94,95].

Ineffective communication can undermine the well-being of providers and patients, the quality of medical services, and the functioning of the organization itself, with vital effects on the relational climate of the organization [96].

According to the literature, organizational efficacy has shown positive influences in several areas of organizational life, including performance, job satisfaction, and well-being [31]. This result is in line with previous studies in the Italian healthcare context [35] that highlighted the relationship between collective efficacy, sense of belonging, job satisfaction, and well-being [44].

The literature suggested that the strains of an asymmetric relationship between doctors and patients, (i.e., not oriented to the active engagement of patients) might eventually deplete clinicians’ emotional resources [88,97]. On the other hand, numerous studies emphasized how patient-centered medicine can benefit the quality of life of physicians and patients [98,99,100]. Even more in the pandemic scenario, the literature [50] showed the crucial role of collaboration between patients/citizens and healthcare professionals during the emergency and that engagement in health management and their attitudes toward preventive measures are functions of the individual’s beliefs.

Consistent with this literature, patient engagement orientation was positively correlated with communication self-efficacy beliefs.

Although orientation towards patient engagement was found to be a protective factor against emotional exhaustion during the COVID-19 pandemic, [49] there was a lack of studies examining its relationship to well-being. Contrary to our hypotheses, orientation towards patient engagement does not appear to be correlated with well-being. Presumably, this result is since other variables mediate the relationship between the two.

The role of communication returns as a factor in promoting well-being, even when looking at outcomes related to the organization’s NTS. The results emphasized that being in an organization capable of developing prompt, effectively accessible, and useful communication is significantly and positively correlated with job satisfaction. A finding that should be considered in light of the pandemic emergency, but also for the future, when healthcare organizations will be asked to reorganize the “new normal”, post-emergency.

Results on situational awareness go in the same direction. Assessing that the organization is capable of promptly understanding the evolution of the risk in the workplace, is associated with employees’ job satisfaction. Thus, the fact that the organization is perceived as capable of implementing valuable strategies to mitigate the risk of contagion in the workplace could increase job satisfaction and well-being.

Finally, in line with previous studies in healthcare [31], experiencing workplace social support and a sense of belonging resulted in the best management of difficulties caused by the pandemic emergency, and in this study represented a significant workplace resource. Therefore, it is plausible to assume that perceiving their organization as supportive and feeling that they can count on colleagues and superiors’ support in this period of emergency, had a positive impact on the perceived well-being of healthcare workers. In terms of the unconfirmed hypotheses, the strong effect of satisfaction probably made the relationship between the variables included in the study and mental well-being statistically non-significant, except for the sense of belonging. Therefore, the mediating role of job satisfaction should be tested in future studies.

Additionally, the role of a sense of belonging to the organization in mental well-being should be emphasized. Sense of belonging is related to positive mental health when it provides the means through which one is integrated into an organization such that he or she feels needed and valued and contributes to the organization in return.

Hence, one of the mechanisms to contribute to healthcare workers’ well-being could be identified in the creation of a sense of belonging and respect, promoting feelings of trust, pride, and cooperation [31,101].

The study has some limitations, including the fact that it is a cross-sectional study, and data were collected using an online questionnaire. Because both exposure and outcome are assessed simultaneously, a true cause-and-effect relationship cannot be established. Moreover, although the sample was adequate for the implemented analyses, it should be noted that it is small and inhomogeneous due to the difficulties of recruiting healthcare workers in the pandemic era [102]. Consequently, this does not allow for comparisons by gender and resulted in a small effect size. In addition, the use of an online self-report instrument can easily expose to the risk of social desirability in answers.

To avoid these issues, future studies with a larger and more representative sample of physicians, as well as the adoption of a mixed-methods design, are required.

Despite such limitations, the strong point of the study is represented by the period of administration of the questionnaire, which, even though it is restricted, refers to a particular historical moment, characterized by profound changes and completely unknown experiences, valid for an initial exploratory survey. In this sense, it would be desirable to continue the study with a longitudinal research design.

More specifically, further follow-up studies are necessary to clarify the effects of the COVID-19 pandemic on doctors’ mental health over time and the organizational variables able to moderate such outcomes. In addition, as suggested by other works [8], most studies conducted in healthcare contexts have focused more on experiences of discomfort without emphasizing the necessary attention to the individual and organizational factors which contribute to well-being.

## 5. Conclusions

Few studies have set out to investigate practitioner well-being [103] from a positive psychology perspective, during the recent pandemic [90]. Moreover, the role of the participants’ individual and organizational resources (e.g., efficacy beliefs) in coping with COVID-19 suggests healthcare managers adopt strategies to promote and sustain physicians’ well-being. Specifically, it seems clear that there is a need to implement adequate interventions to make physicians able to rely on their organizations both during the pandemic and after.

Organizational strategies should also support or strengthen personal resources to foster the contribution of workers in managing COVID-19-related issues. Our findings suggest that these interventions should aim to improve workers’ bond with their organizations and their ability to cope with issues related to communication with patients and colleagues as well as organizations’ efficacy in pursuing their goals into account employees’ safety to the point of fostering their satisfaction about their job and, consequently, their well-being. This is in line with the literature [104] that sustains the importance of maintaining a healthy and employable workforce, which is essential for a sustainable healthcare system.

Such strategies, as suggested by Schrijver et al. [105], should be designed based on the medical specialty, career phase, and workplace characteristics and can consist both of collective (e.g., teambuilding, reducing work hours, support groups) and individual interventions (e.g., mentoring, one-to-one supervision, self-care training) [103].

For worker well-being to be supported during the COVID-19 pandemic, an integrated and participatory approach for the enforcement of the identified procedures has been emphasized. Regarding NTS, our results are the first, to our knowledge, that could allow hospital organizational to reflect on the present situation and to define ad hoc interventions aimed to manage and protect of the health of health workers. Finally, the aforementioned interventions should have a wide range of impacts because they may improve not only physicians’ well-being, but also the quality of medical care and patient satisfaction [106].

## Figures and Tables

**Table 1:** Descriptive analyses.

	Range	M	SD	Cronbach’s Alpha
Mental well-being	1–6	4.01	0.96	0.91
Emotional well-being	1–6	4.10	1.25	0.88
Psychological well-being	1–6	4.49	0.98	0.88
Social well-being	1–6	3.39	1.10	0.80
Job satisfaction	1–7	4.91	1.41	-
Collective efficacy	1–7	5.35	0.90	0.77
Communication self-efficacy	1–5	3.39	0.61	0.86
Orientation towards engagement	1–6	4.30	0.69	0.76
Sense of belonging	1–4	2.74	0.63	0.88
Organizational support	1–10	6.31	1.92	0.76
NTS Communication	1–5	3.53	0.91	0.86
NTS Decision-making	1–5	2.94	1.04	0.94
NTS Situational awareness	1–5	2.98	1	0.91
NTS Fatigue management	1–5	2.46	1.10	0.97
NTS Personal Contribution	1–5	3.88	0.75	0.90

**Table 2:** Correlations among the psychological variables.

Psychological Variables	1	2	3	4	5	6	7	8	9	10	11	12	13	14	15
1. Mental well-being	-														
2. Emotional well-being	0.90 **	-													
3. Psychological well-being	0.88 **	0.72 **	-												
4. Social well-being	0.89 **	0.76 **	0.61 **	-											
5. Job satisfaction	0.60 **	0.67 **	0.42 **	0.57 **	-										
6. Collective efficacy	0.35 **	0.30 **	0.45 **	0.18	0.31 **	-									
7. Communication self-efficacy	0.22	0.19	0.34 **	0.05	0.22	0.25 *	-								
8. Orientation towards engagement	0.01	−0.05	0.13	−0.07	−0.70	0.38 **	0.32 **	-							
9. Sense of belonging	0.50 **	0.49 **	0.31 **	0.55 **	0.50 **	0.14	0.02	−0.07	-						
10. Organizational support	0.22	0.20	0.16	0.23 *	0.20	0.26 *	0.07	0.00	0.49 **	-					
11. NTS Communication	0.32 **	0.34 **	0.19	0.36 **	0.41 **	0.31 **	0.14	0.15	0.52 **	0.60 **	-				
12. NTS Decision-making	0.23 *	0.29 *	0.16	0.18	0.27 *	0.24 *	0.19	0.26 *	0.37 **	0.66 **	0.74 **	-			
13. NTS Situational awareness	0.14	0.20	0.07	0.14	0.17	0.22	0.07	0.21	0.34 **	0.65 **	0.78 **	0.88 **	-		
14. NTS Fatigue management	0.19	0.17	0.13	0.18	0.20	0.24 *	−0.00	0.16	0.30 **	0.50 **	0.54 **	0.69 **	0.74 **	-	
15. NTS Personal Contribution	0.19	0.14	0.17	0.18	0.09	0.22	0.05	0.29 *	0.32 **	0.38 **	0.45 **	0.40 **	0.39 **	0.40 **	-

Note. * *p* < 0.05; ** *p* < 0.01.

**Table 3:** Model 1—Multiple linear regression for physicians’ job satisfaction (*n* = 78).

Job Satisfaction R^2^ = 0.374	B	SE_B_	Β	*t*	*p*
Constant	0.160	1.179		0.136	0.892
Collective efficacy	0.466	0.164	0.298	2.834	0.006
Communication self-efficacy	0.491	0.227	0.214	2.159	0.034
Orientation towards engagement	−0.430	0.213	−0.212	−2.024	0.047
Sense of belonging	1.136	0.238	0.512	4.777	<0.001
Organizational support	−0.105	0.080	−0.143	−1.306	0.196

Note. R^2^ = explained variance; B = unstandardised regression coefficient; SE_B_ = standard error of the coefficient; β = standardized coefficient.

**Table 4:** Model 2—Multiple linear regression for physicians’ well-being (*n* = 78).

Mental Well-Being R^2^ = 0.172	Regression Coefficient	SE	Β	*t*	*p*
Constant	0.263	0.759		0.347	0.730
Collective efficacy	0.218	0.112	0.205	1.957	0.054
Communication self-efficacy	0.162	0.151	0.103	1.073	0.287
Orientation towards engagement	−0.068	0.141	−0.49	−0.483	0.630
Sense of belonging	0.477	0.176	0.315	2.716	0.008
Organizational support	−0.035	0.052	−0.071	−0.678	0.500
Job satisfaction	0.253	0.076	0.370	3.338	0.001

Note. R^2^ = explained variance; B = unstandardised regression coefficient; SE_B_ = standard error of the coefficient; β = standardized coefficient.

**Table 5:** Model 3—NTS, Multiple linear regression for physicians’ job satisfaction (*n* = 78).

Job Satisfaction R^2^ = 0.114	Regression Coefficient	SE	Β	*t*	*p*
Constant	3.351	0.785		4.269	<0.001
NTS Communication	1.114	0.260	0.719	4.291	<0.001
NTS Decision making	0.490	0.294	0.362	1.668	0.100
NTS Situational awareness	−1.127	0.352	−0.800	−3.201	0.002
NTS Fatigue management	0.268	0.195	0.209	1.370	0.175
NTS Personal Contribution	−0.284	0.215	−0.151	−1.322	0.190

Note. R^2^ = explained variance; B = unstandardised regression coefficient; SE_B_ = standard error of the coefficient; β = standardized coefficient.

**Table 6:** Model 4—NTS, Multiple linear regression for physicians’ mental well-being (*n* = 78).

Job Satisfaction R^2^ = 0.285	Regression Coefficient	SE	Β	*t*	*p*
Constant	1.424	0.560		2.544	0.013
NTS Communication	0.110	0.185	0.104	0.594	0.555
NTS Decision making	0.067	0.191	0.072	0.350	0.727
NTS Situational awareness	−0.184	0.240	−0.191	−0.769	0.445
NTS Fatigue management	0.062	0.126	0.071	0.491	0.625
NTS Personal Contribution	0.143	0.138	0.112	1.037	0.303
Job satisfaction	0.376	0.075	0.550	5.011	<0.001

Note. R^2^ = explained variance; B = unstandardised regression coefficient; SE_B_ = standard error of the coefficient; β = standardized coefficient.

## Data Availability

The data presented in this study are available on request from the corresponding author.
